# P. gingivalis-LPS Activates the Alzheimer’s Disease-Associated Amyloid Secretase Pathway via Caspase-4

**DOI:** 10.1093/geroni/igaf122.2224

**Published:** 2025-12-31

**Authors:** Ambika Verma, Gohar Azhar, Pankaj Patyal, Xiaomin Zhang, Jeanne Wei

**Affiliations:** University of Arkansas for Medical Sciences, Little Rock, Arkansas, United States; University of Arkansas for Medical Sciences, Little Rock, AR, LITTLE ROCK, Arkansas, United States; University of Arkansas for Medical Sciences, Little Rock, Arkansas, United States; University of Arkansas for Medical Sciences, Little Rock, Arkansas, United States; University of Arkansas for Medical Sciences, Little Rock, AR, LITTLE ROCK, Arkansas, United States

## Abstract

Porphyromonas gingivalis, a periodontal pathogen, has been implicated in the pathogenesis of Alzheimer’s disease (AD) and related dementias (ADRD) through its virulent lipopolysaccharide (LPS) component. Amyloid pathologies, characterized by the accumulation of amyloid-beta (Aβ), are central features of AD/ADRD. Our previous work demonstrated that P. gingivalis-LPS treatment activated the amyloid precursor protein (APP) and leading to the production of Aβ1-42 and Aβ1-40 peptides in SH-SY5Y cells. Caspase-4, an inflammatory mediator that directly senses and binds to cytosolic LPS, plays a critical role in inflammatory signaling. However, the involvement of caspase-4 in P. gingivalis-LPS induced amyloid pathologies remains underexplored. We hypothesized that P. gingivalis-LPS contributes to AD/ADRD pathogenesis by activating the Alzheimer’s disease-associated amyloid secretase pathway mediated by caspase-4. To investigate this, we performed caspase-4 knockdown in SH-SY5Y cells using caspase-4 specific siRNA and the caspase-4 inhibitor Ac-LEVD-CHO. Presenilin-1 (PS1), a key γ-secretase component, is essential for amyloidogenic APP processing and Aβ1-42 production, was also examined. Our findings demonstrated that P. gingivalis-LPS treatment activated both APP and PS1 in SH-SY5Y cells, with caspase-4 silencing significantly reducing the expression of these proteins compared to negative control siRNA (p < 0.05). Furthermore, P. gingivalis-LPS induced elevated Aβ1-42 and Aβ1-40 peptide release was reversed upon treatment with caspase-4 siRNA or Ac-LEVD-CHO (p < 0.05). These results provide compelling evidence that caspase-4 mediates P. gingivalis-LPS induced neuroinflammation and highlight the complex interactions between caspase-4, APP, PS1 and Aβ in the pathogenesis of AD/ADRD.

